# A COVID-19 mortality prediction model for Korean patients using nationwide Korean disease control and prevention agency database

**DOI:** 10.1038/s41598-022-07051-4

**Published:** 2022-02-28

**Authors:** Yongho Jee, Yi-Jun Kim, Jongmin Oh, Young-Ju Kim, Eun-Hee Ha, Inho Jo

**Affiliations:** 1 Advanced Biomedical Research Institute, Ewha Womans University Seoul Hospital, Seoul, Republic of Korea; 2 Institute of Convergence Medicine, Ewha Womans University Mokdong Hospital, Seoul, Republic of Korea; 3 Department of Environmental Medicine, Ewha Womans University College of Medicine, Seoul, Republic of Korea; 4 Department of Obstetrics and Gynecology, Ewha Womans University College of Medicine, Seoul, Republic of Korea; 5 Department of Molecular Medicine, College of Medicine, Ewha Womans University, Seoul, Republic of Korea

**Keywords:** Medical research, Epidemiology

## Abstract

The experience of the early nationwide COVID-19 pandemic in South Korea led to an early shortage of medical resources. For efficient resource allocation, accurate prediction of the prognosis or mortality of confirmed patients is essential. Therefore, the aim of this study was to develop an accurate model for predicting COVID-19 mortality using epidemiolocal and clinical variables and for identifying a high-risk group of confirmed patients. Clinical and epidemiolocal variables of 4049 patients with confirmed COVID-19 between January 20, 2020 and April 30, 2020 collected by the Korean Disease Control and Prevention Agency were used. Among the 4049 total confirmed patients, 223 patients died, while 3826 patients were released from isolation. Patients who had the following risk factors showed significantly higher risk scores: age over 60 years, male sex, difficulty breathing, diabetes, cancer, dementia, change of consciousness, and hospitalization in the intensive care unit. High accuracy was shown for both the development set (n = 2467) and the validation set (n = 1582), with AUCs of 0.96 and 0.97, respectively. The prediction model developed in this study based on clinical features and epidemiological factors could be used for screening high-risk groups of patients and for evidence-based allocation of medical resources.

## Introduction

Coronavirus disease-2019 (COVID-19) has become a global pandemic that is threatening far more than a health crisis. It also affects societies and economics^1–3^. As the number of confirmed patients has explosively increased, there is a need for risk stratification both for preventing (i.e., home quarantine, social distancing) and for treating confirmed patients (i.e., hospitalization vs. community isolation). Although the COVID-19 pandemic has caused more than 5,000,000 death worldwide, there are also a significant number of asymptomatic patients who become infected and recover asymptomatically^4^. Identification of high-risk confirmed patients is required to allow better allocation of existing available medical resources. According to the Centers for Disease Control and Prevention (CDC), confirmed patients who are over 65 years old, who live in nursing homes, and who have at least one of the following conditions, chronic lung disease, serious heart conditions, severe obesity, diabetes, liver disease, and immunocompromised status, are at a high risk of death due to COVID-19^4^. Although the CDC guideline has been used as a reference for overall patients, more precise prediction using patient multivariable data is required to evaluate individualized risk and to establish evidence for risk stratification^5^. In this context, an accurate model for predicting COVID-19 mortality and identifying risk factors could help stratify management strategies for patients who have a high risk of death. Previous mortality prediction studies reported in the early period of COVID-19 pandemic used relatively few variables and showed lower predictive performance^6^.

Since the COVID-19 outbreak started in Hubei Province of the People’s Republic of China, the majority of early prediction studies were based on Chinese data^7–10^.

Barda et al. established a prediction model by combining the development of a baseline respiratory infection risk predictor and a postprocessing method using Israel data^11^. However, since they did not have individual data, they were not able to test its prediction performance. According to Wynants’ review of early reported prediction models regarding COVID-19, these proposed models are poor with a high risk of bias due to the lack of external validation of models^5^.

Since South Korea is geopolitically close to China, it is one of the countries most affected by COVID-19 during the early stage of the pandemic. In reality, Korea experienced an explosive outbreak in the first two months since the first confirmed patient was detected on January 20^12^. A mortality prediction model using a machine method based on sociodemographic and medical information of national health insurance data has been proposed^13^. However, it was focused on socioeconomic variables as predictors rather than clinical and epidemiological factors. Clinical experience and epidemiological characteristics have been reported as major factors associated with heterogeneity of prognosis after COVID-19 confirmation^14^. Therefore, the aim of this study was to establish a COVID-19 mortality prediction model using clinical and epidemiological variables nationally collected by Central Disease Control Headquarters.

## Results

### Baseline characteristics

Since the first patient was confirmed with COVID-19 on January 20, 2020, 4049 patients were managed by the government database and released from quarantine or death until April 30, 2020. Among 4049 released patients, the case mortality was 5.51% (223 deaths and 3826 recoveries).

We compared the distribution of patients according to epidemiological and clinical characteristics. We also conducted a logistic regression analysis for mortality outcome by unadjusting (univariable) or adjusting (multivariable) covariates. Results are shown in Table 1. In univariable analysis, age over 40, male sex, runny nose, and headache significantly increased the risk of mortality, while having abnormal changes in consciousness (ACC), diabetes, hypertension, cancer history, dementia, and hospitalization in the intensive care unit was protective. In the multivariable analysis after adjusting for covariates, age over 40 years and having a runny nose remained significant risk factors for mortality. Protective variables remained protective after adjusting for covariates.

**Table 1:** Clinical and epidemiological characteristics of COVID-19 confirmed patients according to outcome status: categorical variable.

		Quarantine release N (%)	Death N (%)	Unadjusted OR (95% CI)	Adjusted OR (95% CI)
Age (sex adjusted)	0–39	1144 (99.83%)	2 (0.17%)	1.0	1.0
	40–49	499 (99.60)	2 (0.40)	2.60 (0.37–18.55)	2.67 (0.22–33.09)
	50–59	839 (98.36)	14(1.64)	10.40 (2.35–45.90)	5.51 (0.67–45.38)
	60–69	732 (96.19)	29 (3.81)	23.49 (5.58–98.78)	8.48 (1.07–67.08)
	70–79	418 (86.36)	66 (13.64)	94.60(23.05–88.30)	17.61 (2.25–138.14)
	≥ 80	194 (63.82)	110 (36.18)	385.55 (94.09–999)	75.01(9.54–589)
Sex (age adjusted)	Men	1454 (92.73)	114 (7.27)	1.0	1.0
	Women	2372 (95.61)	109 (4.39)	0.45 (0.34–0.61)	0.54 (0.36–0.82)
Fever	Yes	849 (90.8)	86 (9.20)	1.0	1.0
	No	2977 (95.6)	137 (4.40)	0.37 (0.27–0.52)	0.73 (0.42–1.27)
Runny nose	Yes	386 (98.47)	6 (1.53)	1.0	1.0
	No	3440 (94.07)	217 (5.93)	2.87 (1.22–6.74)	2.95 (1.09–7.99)
SOB	Yes	453 (80.75)	108 (19.25)	1.0	1.0
	No	3373 (96.70)	115 (3.30)	0.22 (0.16–0.30)	0.38 (0.25–0.58)
Headache	Yes	699 (98.45)	11 (1.55)	1.0	1.0
	No	3127 (93.65)	212 (6.35)	2.41 (1.28–4.57)	2.20 (1.02–4.74)
ACC	Yes	10 (31.25)	22 (68.75)	1.0	1.0
	No	3816 (95.00)	201 (5.00)	0.04 (0.01–0.10)	0.07 (0.02–0.21)
Diabetes	Yes	512 (84.91)	91 (15.09)	1.0	1.0
	No	3314 (96.17)	132 (3.83)	0.49 (0.36–0.66)	0.47 (0.31–0.72)
Hypertension	Yes	889 (86.90)	134 (13.10)	1.0	1.0
	No	2937 (97.06)	89 (2.94)	0.65 (0.47–0.89)	0.99 (0.66–1.50)
Heart failure	Yes	37 (69.81)	16 (30.19)	1.0	1.0
	No	3789 (94.82)	207 (5.18)	0.52 (0.27–1.03)	0.89 (0.36–2.21)
CKD	Yes	31 (65.96)	16 (34.04)	1.0	1.0
	No	3795 (94.83)	207 (5.17)	0.24 (0.11–0.52)	0.85 (0.44–1.67)
Cancer history	Yes	113 (83.70)	22 (16.30)	1.0	1.0
	No	3713 (94.86)	201 (5.14)	0.38 (0.22–0.66)	0.28 (0.13–0.57)
Dementia	Yes	138 (65.09)	74 (34.91)	1.0	1.0
	No	3688 (96.12)	149 (3.88)	0.40 (0.27–0.59)	0.21 (0.13–0.35)
Sickbed	Yes	95 (55.23)	77 (44.77)	1.0	1.0
	No	3731 (96.23)	146 (3.77)	0.08 (0.05–0.12)	0.13 (0.08–0.21)

*SOB* shortness of breath, *ACC* Abnormal change of consciousness, *CKD* chronic kidney disease.

### Factors associated with mortality from COVID‑19

Table 2 summarizes differences in clinical characteristics for continuous variables and the risk of COVID-19 mortality by 1-unit increase of each clinical variable. Heart rate intensity (OR 1.03, 95% CI 1.02–1.04) and temperature (OR 1.94, 95% CI 1.55–2.43) were associated with an increased risk of COVID-19 mortality. Higher levels of hemoglobin, hematocrit, and lymphocytes were associated with a significantly lower risk of mortality. Based on the exploratory analysis results shown in Tables 1 and 2, a prediction model for the development set was established, as shown in Table 3. The odds ratio (regression coefficient) of mortality risk was determined to produce a risk score.

**Table 2:** Clinical and epidemiological characteristics of COVID-19 confirmed patients according to outcome status: continuous variable.

	Quarantine release Mean (standard deviation)	Death Mean (standard deviation)	OR (95% CI)
Systolic blood pressure	2.77 ± 1.33	2.99 ± 1.47	0.90 (0.80–1.01)
Diastolic blood pressure	2.01 ± 0.97	1.89 ± 1.01	0.91 (0.78–1.06)
Heart rate intensity	85.66 ± 15.00	89.40 ± 19.93	1.03 (1.02–1.04)
Temperature	36.92 ± 0.57	37.10 ± 0.76	1.94 (1.55–2.43)
hemoglobin (G/DL)	13.37 ± 1.69	11.76 ± 2.21	0.76 (0.69–0.82)
hematocrit (%)	39.51 ± 4.71	34.95 ± 6.68	0.91 (0.89–0.94)
Lymphocyte (%)	29.96 ± 11.18	15.34 ± 11.06	0.90 (0.88–0.92)

**Table 3:** Mortality prediction equation: logistic model (equation using development set:)

	Development set (N = 2467) beta (se)
Intercept	20.3083 (2.6748)
Age 60–69	0.9596 (0.4714)
Age 70–79	1.4935 (0.4542)
Age ≥ 80	3.3010 (0.4538)
Men	− 0.7845 (0.2828)
Fever	− 0.8813 (0.2765)
shortness of breath	− 0.9160 (0.2848)
Abnormal change of consciousness	− 2.9806 (0.8839)
Dementia	− 0.6318 (0.2689)
Cancer	− 1.1150 (0.4675)
Dementia	− 1.5940 (0.3426)
sickbed	− 2.6019
Hematocrit	− 0.0767 (0.0238)
Lymphocyte	− 0.0694 (0.0131)

COVID 19 Mortality = 0.9596*(Age 60–69) + 1.4935*(Age 70–79) + 3.3010*(Age ≥ 80) − 0.7845*(Sex)  − 0.8813*(Fever)  − 0.9160*(SOB)  − 2.9806*(ACC)  − 0.6318*(Diabetes)  − 1.1150*(Malignancy)  − 1.5940*(Dementia)  − 2.0619*(Sickbed type)  − 0.0767*(hematocrit)  − 0.0694*(Lymphocyte).

### Performance of prediction model

**Figure 1:**
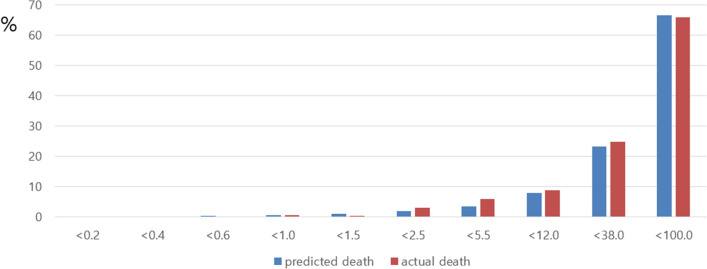
Probability of Predicted and Actual Deaths in total set of participants.

**Figure 2:**
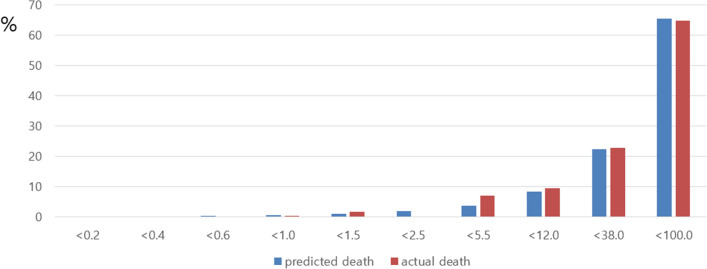
Probability of Predicted and Actual Deaths in development set.

**Figure 3:**
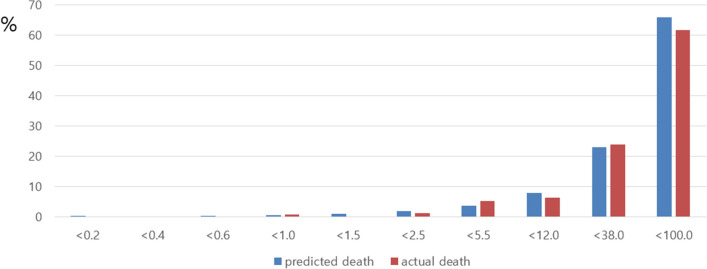
Probability of Predicted and Actual Deaths in validation set.

**Figure 4:**
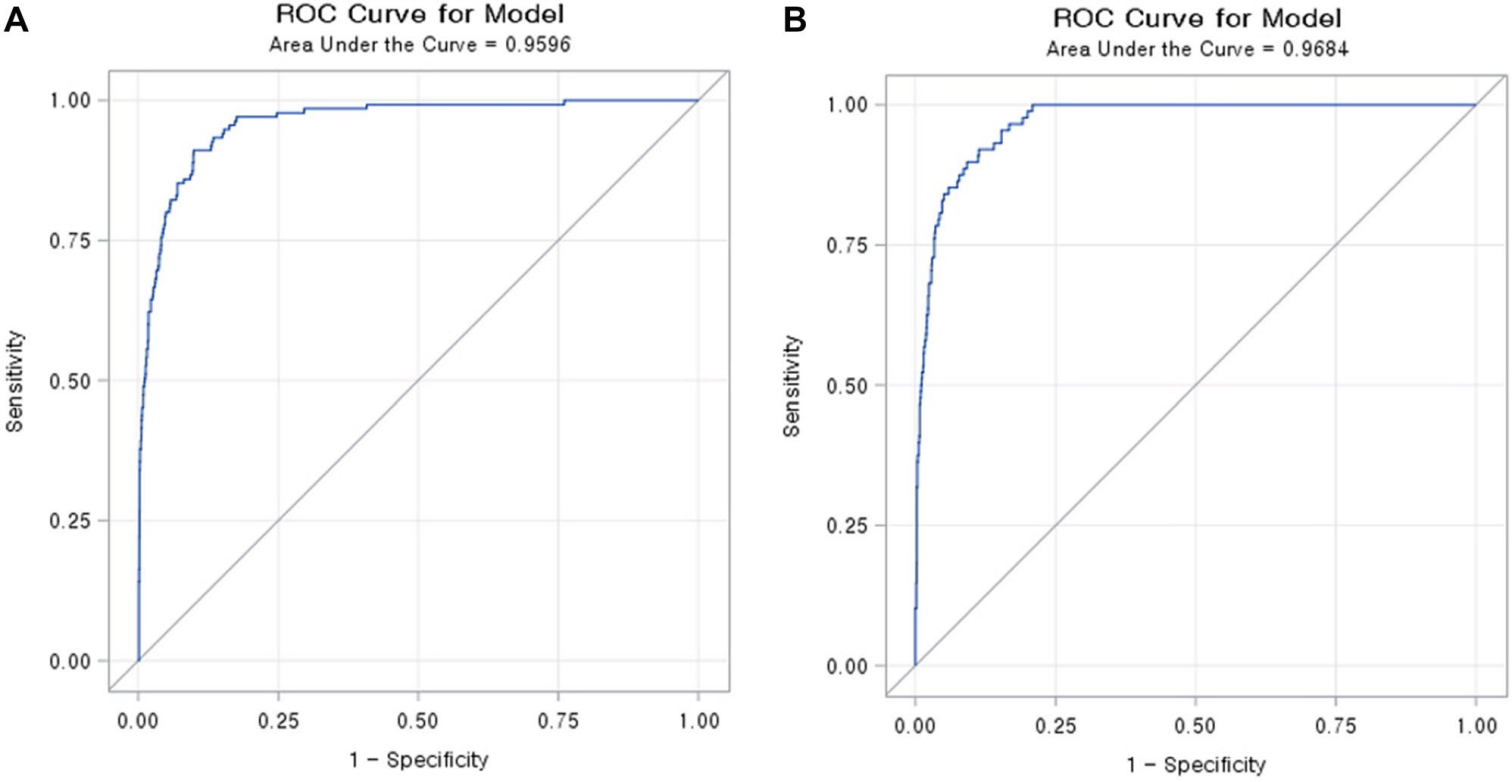
Performance of the mortality prediction models on development set (**A**) and validation set (**B**).

We applied our risk score to our total set, the development set, and the validation set. Figures 1, 2, 3 show comparison results between the predicted mortality and the actual mortality by risk score stratified by decile. Figure 1 shows the results for the total set of participants. Figure 2 describes results for the development set. Figure 3 shows results for the validation set. The performance of each dataset was evaluated using ROC curves. Results are shown in Fig. 4. Our prediction model showed good performance for both the development set and the validation set, with areas under the curve of 0.9656 and 0.9684, respectively.

## Discussion

Our study developed and validated a COVID-19 mortality prediction model based on clinical and epidemiological data of COVID-19 4049 confirmed patients recruited by Korea Centers for Disease Control and Prevention. The high AUC value of 0.9684 indicated the good reliability and performance of our model. The course of clinical symptoms of coronavirus ranges from asymptomatic infection to acute respiratory distress (ARDS) and death. As the period of the COVID-19 global pandemic lasts longer, a shortage of medical resources comes earlier. Therefore, differentiated patient management based on evidence is required. Risk stratification also suggests evidence to allocate resources efficiently when medical resources are limited^4^. Several previous Korean studies have reviewed the characteristics of mortality cases of COVID-19. The Korean Society of Infectious Diseases and Korea Centers for Disease Control and Prevention has analyzed 54 COVID-19 mortality cases since the first mortality occurred from February 19 to March 10, 2020. The median age of mortality cases was 75.5 years. Of all mortality cases, 61.1% were men. The majority of such patients also had various underlying diseases, such as hypertension, heart disease, diabetes, dementia, and stroke. Another study reported in Korea focused on 20 mortality cases in Gyeongbuk Province and Daegu city, where the second outbreak wave occurred in February based on medical chart review^15^. Average age of mortality cases was 72 years. Of these mortality cases, 55.1% were women, and 74.5% had an underlying disease. The median length from hospitalization to death was 8 days. Comorbidities such as diabetes, chronic lung disease, and chronic neurologic disease were significant risk factors associated with COVID-19 mortality. Clinical manifestations observed before death were abnormal heart rate intensity, systolic blood pressure, respiratory rate, oxygen saturated by pulse oximetry on room air, and altered mental status^16^. Although these two studies reported the clinical characteristics of the deceased in detail at the level of descriptive epidemiology, which contributed to the overall understanding of COVID-19 patients, their numbers of cases were relatively small and were not enough for associational inference. One study developed an evidence-based COVID-19 prognostic model for military personnel in Korea^17^. Although there was a problem of generalization since it was developed for soldiers, age, body temperature, physical activity, history of cardiovascular disease, hypertension, visit to a region with an outbreak, feverishness, dyspnea, lethargy, and symptoms of chills were reported as significant predictors (overall C statistic: 0.963; 95% CI: 0.936–0.99)^17^.

Machine learning based COVID-19 mortality prediction on Korean population was reported by several studies^13,18^.

An et al. developed a COVID-19 mortality prediction model using machine learning after recruiting 10,237 COVID-19 confirmed patients and 228 mortality cases between January 20, 2020 and April 16, 2020^13^. This prediction model used various variables, including socioeconomic status linked with National Health Insurance Service. However, specific clinical and epidemiological variables were lacking since that study was focused on the linkage with NHIS data. For mortality prediction, LASSO and linear SVM were used in that study, with AUC values of 0.963 and 0.962, respectively. The most significant factors in the mortality prediction model using LASSO were old age, preexisting DM, and cancer. The most significant factors in random forest were old age, infection route (cluster infection or infection from personal contact), and underlying hypertension^13^. However, that model could not be immediately applied to the field or clinics due to the lack of specific clinical variables.

Das et al. also aimed to predict mortality among confirmed COVID-19 patients in South Korea using machine learning and deploy the best performing algorithm as an open-source online prediction tool for decision-making. They found that the logistic regression algorithm was the best performer in terms of discrimination^18^. Oh et al. aimed to investigate whether comorbid musculoskeletal disorders (MSD)s and pain medication use was associated with in-hospital mortality among patients with COVID-19. They found MSDs were not associated with increased in-hospital mortality among patients with COVID-19^19^. Lee et al. found potential associations between physical activity and risk of infection, severe illness from COVID-19 and COVID-19 mortality using a nationwide cohort from South Korea^20^.

Previous foreign studies have reported that different clinical experiences can lead to substantial heterogeneity in the prognostic trajectory of COVID-19 confirmed patients spanning from patients who are asymptomatic to those with mild, moderate, and severe disease forms with low survival rates^21,22^. A COVID-19 mortality prediction model was developed previously by analyzing data from 3841 confirmed patients in New York, USA recruited from March 9 to April 6, 2020 using machine learning^21^. Sex, age, race, oxygen saturation, COPD, hypertension, and diabetes were found to be significant variables in that model, with AUCs of 0.91 to 0.94. However, blood test results were not included in that model. In that study, the minimum oxygen saturation was emphasized as a central factor in mortality prediction^22^.

A prediction model was developed after analyzing 53,001 ICU patients requiring mechanical ventilation as well as those diagnosed with pneumonia from the US Medical Information Mart for Intensive Care (MIMIC). When that model was applied to 114 confirmed COVID-19 patients^23^, the AUCs for 12, 24, 48, and 72 h were reported to be 0.82, 0.81, 0.77, and 0.75, respectively^23^. Our study probably used the largest data set up to date to predict COVID-19 mortality involving specific clinical features of COVID-19 patients in Korea. The main advantage of our study was that we collected large range of clinical and epidemiological variables at the time when patient was enrolled as a confirmed case. The results were obtained after a certain period of health system encounter or immediately after the diagnosis of COVID-19. Although we merely conducted logistic regression analysis, both the development and validation sets showed high areas under the curve (0.9656 and 0.9684, respectively). although there have been studies with larger sample sizes or extensive data collections, they had difficulty on interpretation on the results due to lack of algorithm.

Moreover, our model has the advantage of being able to easily interpret factors associated with the high mortality rate of individuals according to the detailed algorithm shown in the model. In that context, our model has high practical value for risk stratification in the clinical field.

The main limitation of our study was the issue of validation. Although our dataset was relatively large and involved specific clinical features, we merely conducted internal validation due to the lack of a dataset that had similar sizes and variables in Korea. Thus, the possibility of overestimation exists, which requires cautious interpretation of our results.

However, in terms of Personal Information Protection issues, current COVID-19 mortality data in Korea is merely collected and managed by the Government agency called Korean Disease Control and Prevention Agency (KDCA). Thus, no other dataset was available in Korea rather than the KDCA dataset. Thus, an external validation study using data from COVID-19 patients that occurred afterwards is required in the future.

## Subjects and methods

### Study population

Our study was based on the dataset established by Korean Disease Control and Prevention Agency Central Disease Countermeasure Headquarters. Individual-level data for 4049 COVID-19 patients whose quarantine release was confirmed among patients infected between January 20, 2020 and April 30, 2020 were collected. Complete nationwide inpatient and outpatient data of patients who visited any medical institution with a confirmed diagnosis of COVID-19 during the study period were obtained. The definition of COVID-19 confirmation was determined by positive PCR-based clinical laboratory testing for SARS-CoV-2. Personal information deidentification measures were applied in accordance with governmental guidelines for nonidentification measures and proceeded in accordance with adequacy evaluation.

### Risk factor measurement

The collected data used in our study included 41 variables categorized into seven subtypes as follows: (1) basic data (age, sex, death/quarantine released, length of stay between infection and death/quarantine released, pregnancy), (2) body index (height, weight), (3) initial examination finding (systolic/diastolic blood pressure, heart rate, body temperature), (4) clinical findings at hospitalization (history of fever, cough, sputum production, sore throat, runny nose/rhinorrhea, muscle aches/myalgia, fatigue/malaise, shortness of breath/dyspnea, headache, altered consciousness/confusion, vomiting/nausea, diarrhea), (5) comorbidity and past history (diabetes, hypertension, heart failure, chronic heart condition, asthma, chronic obstructive pulmonary disease, chronic kidney failure, cancer, chlimitaronic hepatic disease, rheumatism, autoimmune disease, dementia), (6) sickbed type and clinical severity, and (7) complete blood cell count. Each variable was either self-reported or recorded by professional health care providers. Mortality was defined when a patient with COVID-19 died during their encounter with the health system during the study period (January 1, 2020 ~ April 30, 2020). The data usage and study design of our study were approved by the Institutional Review Board of Ewha Womans University Seoul Hospital, and informed consent was obtained from each subject (SEUMC 2020-09-009). All methods were performed in accordance with relevant guidelines and regulations.

### Statistical analysis

Risk scores for our COVID-19 mortality prediction model were developed by logistic regression analysis. We stratified our data into two groups: 60% random sampling (development set data) for model development and the remaining 40% (test data set) for internal validation. All analyses were conducted using SAS version 9.4 (SAS Institute Inc., Cary, NC, USA).
